# Supplementary material for: Lineage-specific intersection of endothelin and GDNF signaling in enteric nervous system development
Source: eLife. 2024 Dec 6;13:RP96424. doi: 10.7554/eLife.96424 (PMC11623925; doi:10.7554/eLife.96424)
Supplement: Figure 1—source data 1. [file elife-96424-fig1-data1.docx]

**Figure 1b source data**

|  | BW(g) | | | |
| --- | --- | --- | --- | --- |
| litter ID | control | EdnrbKO | cKO(Pax2Cre) | cKO(Wnt1Cre) |
|  | n=21 | n=12 | n=7 | n=6 |
| 1 | 16.20 | 11.10 |  |  |
|  | 17.60 | 11.20 |  |  |
|  |  | 10.70 |  |  |
|  |  | 12.70 |  |  |
| 2 | 14.20 | 9.70 |  |  |
|  | 15.60 |  |  |  |
| 3 | 15.90 | 9.51 |  |  |
|  |  | 11.76 |  |  |
| 4 | 16.50 | 9.70 |  |  |
|  | 14.50 | 9.40 |  |  |
|  | 14.50 | 9.20 |  |  |
| 5 | 17.08 | 6.91 |  |  |
|  | 17.53 | 9.51 |  |  |
| 6 | 19.10 |  |  | 7.10 |
|  | 20.30 |  |  | 7.80 |
| 7 | 11.04 |  |  | 7.90 |
|  |  |  |  | 8.70 |
| 8 | 13.60 |  |  | 11.00 |
|  |  |  |  | 10.63 |
| 9 | 9.90 |  | 6.60 |  |
| 10 | 9.94 |  | 6.07 |  |
|  | 11.32 |  | 9.21 |  |
| 11 | 13.39 |  | 7.52 |  |
|  | 12.15 |  | 10.84 |  |
| 12 | 14.80 |  | 12.67 |  |
|  | 14.80 |  | 12.38 |  |

**Figure 1c source data**

| dried fecal weight (mg) | | | |
| --- | --- | --- | --- |
| control | EdnrbKO | cKO(Pax2Cre) | cKO(Wnt1Cre) |
| 684.0 | 0.0 | 0.0 | 0.0 |
| 649.0 | 0.0 | 0.0 | 0.0 |
| 980.0 | 0.0 | 0.0 | 0.0 |
| 830.0 | 0.0 | 12.7 | 0.0 |
| 900.0 | 0.0 | 10.0 | 0.0 |
| 682.0 | 0.0 | 0.8 | 0.0 |
