# Supplementary material for: Lineage-specific intersection of endothelin and GDNF signaling in enteric nervous system development
Source: eLife. 2024 Dec 6;13:RP96424. doi: 10.7554/eLife.96424 (PMC11623925; doi:10.7554/eLife.96424)
Supplement: Figure 2—source data 1. [file elife-96424-fig2-data1.docx]

**Figure 2v-x source data**

|  | Pax2Cre | | | | | | | Pax2Cre/Ret | | | | |
| --- | --- | --- | --- | --- | --- | --- | --- | --- | --- | --- | --- | --- |
| litter  ID | # GFP^+^  (%) | # GFP^+^/Ret^+^  (%) | | # GFP^-^/Ret^+^  (%) | | # total | # GFP^+^/Ret^-^  (%) | # GFP^+^  (%) | # GFP^+^/Ret^+^  (%) | # GFP^-^/Ret^+^  (%) | # total | # GFP^+^/Ret^-^  (%) |
| 1 | 37 (16.1) | 4 (1.7) | | 193 (83.9) | | 230 | 33 (14.4) | 14 (11.6) | 0 (0) | 107 (88.4) | 121 | 14 (11.6) |
| 2 | 27 (9.2) | 3 (1.0) | | 266 (90.8) | | 293 | 24 (8.2) | 12 (6.8) | 0 (0) | 165 (93.2) | 177 | 12 (6.8) |
|  | 30 (10.3) | 4 (1.4) | | 262 (89.7) | | 292 | 26 (8.9) | 19 (10.3) | 0 (0) | 166 (89.7) | 185 | 19 (10.3) |
| 3 | 28 (10.8) | 5 (1.9) | | 231 (89.2) | | 259 | 23 (8.9) | 12 (7.1) | 0 (0) | 156 (92.9) | 168 | 12 (7.1) |
|  | 33 (14.5) | 4 (1.8) | | 194 (85.5) | | 227 | 29 (12.8) | 13 (8.5) | 0 (0) | 140 (91.5) | 153 | 13 (8.5) |
|  |  |  | |  | |  |  | 17 (11.7) | 0 (0) | 128 (88.3) | 145 | 17 (11.7) |
| 4 | 19 (9.6) | 4 (2.0) | | 178 (90.4) | | 197 | 15 (7.6) | 7 (6.4) | 0 (0) | 103 (93.6) | 110 | 7 (6.4) |
|  |  |  | |  | |  |  | 14 (12.1) | 0 (0) | 102 (87.9) | 116 | 14 (12.1) |
|  |  |  | |  | |  |  |  |  |  |  |  |
|  | Wnt1Cre | | | | | | | Wnt1Cre/Ret | | | | |
| Litter  ID | # GFP^+^  (%) | | # GFP^+^/Ret^+^  (%) | | # GFP^-^/Ret^+^  (%) | # total | # GFP^+^/Ret^-^  (%) | # GFP^+^  (%) | # GFP^+^/Ret^+^  (%) | # GFP^-^/Ret^+^  (%) | # total | # GFP^+^/Ret^-^  (%) |
| 1 | 204 (98.6) | | 172 (83.1) | | 3 (1.4) | 207 | 32 (15.5) | 0 (0) | 0 (0) | 0 (0) | 0 |  |
|  | 205 (97.6) | | 176 (83.8) | | 5 (2.4) | 210 | 29 (13.8) | 0 (0) | 0 (0) | 0 (0) | 0 |  |
| 2 | 146 (98.0) | | 132 (88.6) | | 3 (2.0) | 149 | 14 (9.4) | 0 (0) | 0 (0) | 0 (0) | 0 |  |
|  | 149 (97.4) | | 126 (82.4) | | 4 (2.6) | 153 | 23 (15.0) | 0 (0) | 0 (0) | 0 (0) | 0 |  |
| 3 | 189 (97.4) | | 154 (79.4) | | 5 (2.6) | 194 | 35 (18.0) | 0 (0) | 0 (0) | 0 (0) | 0 |  |
| 4 | 157 (98.1) | | 142 (88.8) | | 3 (1.9) | 160 | 15 (9.4) | 0 (0) | 0 (0) | 0 (0) | 0 |  |

**Figure 2y-z source data**

|  | Pax2Cre | | | | | Pax2Cre/Ret | | | | |
| --- | --- | --- | --- | --- | --- | --- | --- | --- | --- | --- |
| Litter  ID | # GFP^+^  (%) | # GFP^+^/Ret^+^  (%) | # GFP^-^/Ret^+^  (%) | # total | # GFP^+^/Ret^-^  (%) | # GFP^+^  (%) | # GFP^+^/Ret^+^  (%) | # GFP^-^/Ret^+^  (%) | # total | # GFP^+^/Ret^-^  (%) |
| 1 | 590 (72.4) | 130 (16.0) | 225 (27.6) | 815 | 460 (56.4) | 252 (87.5) | 0 (0) | 36 (12.5) | 288 | 252 (87.5) |
| 2 | 542 (71.8) | 178 (23.6) | 213 (28.2) | 755 | 364 (48.21) | 235 (78.3) | 0 (0) | 65 (21.7) | 300 | 235 (78.3) |
| 3 | 569 (66.7) | 228 (26.7) | 283 (33.3) | 852 | 341 (40.1) | 197 (75.8) | 0 (0) | 63 (24.2) | 260 | 197 (75.8) |
| 4 | 654 (69.9) | 270 (28.9) | 282 (30.1) | 936 | 384 (41.0) | 213 (67.4) | 0 (0) | 155 (32.6) | 474 | 213 (67.4) |
| 5 | 376 (48.6) | 295 (38.2) | 397 (51.4) | 773 | 81 (10.5) | 186 (71.0) | 0 (0) | 76 (29.0) | 262 | 186 (71.0) |
|  |  |  |  |  |  | 96 (42.2) | 0 (0) | 132 (57.8) | 228 | 96 (42.2) |
|  |  |  |  |  |  |  |  |  |  |  |
|  | Wnt1Cre | | | | | Wnt1Cre/Ret | | | | |
| Litter  ID | # GFP^+^  (%) | # GFP^+^/Ret^+^  (%) | # GFP^-^/Ret^+^  (%) | # total | # GFP^+^/Ret^-^  (%) | # GFP^+^  (%) | # GFP^+^/Ret^+^  (%) | # GFP^-^/Ret^+^  (%) | # total | # GFP^+^/Ret^-^  (%) |
| 1 | 555 (78.7) | 405 (57.5) | 150 (21.3) | 705 | 150 (21.3) | 0 (0) | 0 (0) | 0 (0) | 0 |  |
| 2 | 934 (92.6) | 535 (54.7) | 43 (4.4) | 977 | 400 (40.9) | 0 (0) | 0 (0) | 0 (0) | 0 |  |
| 3 | 884 (93.4) | 497 (52.5) | 62 (6.6) | 946 | 387 (40.9) | 0 (0) | 0 (0) | 0 (0) | 0 |  |
|  | 920 (94.1) | 564 (57.7) | 58 (5.9) | 978 | 356 (36.4) |  |  |  |  |  |
| 4 | 698 (59.0) | 508 (42.9) | 486 (41.1) | 1184 | 190 (16.1) | 0 (0) | 0 (0) | 0 (0) | 0 |  |
| 5 | 588 (67.4) | 364 (41.7) | 284 (32.6) | 872 | 224 (25.7) | 0 (0) | 0 (0) | 0 (0) | 0 |  |
