# Supplementary material for: Lineage-specific intersection of endothelin and GDNF signaling in enteric nervous system development
Source: eLife. 2024 Dec 6;13:RP96424. doi: 10.7554/eLife.96424 (PMC11623925; doi:10.7554/eLife.96424)
Supplement: Figure 3—source data 1. [file elife-96424-fig3-data1.docx]

**Figure 3p source data**

|  | CGRP+ ave. px area/crypt | | | |
| --- | --- | --- | --- | --- |
| litter ID | control | EdnrbKO | cKO(Pax2Cre) | cKO(Wnt1Cre) |
| 1 | 4.800 | 0.013 |  |  |
| 1 |  | 0.087 |  |  |
| 2 | 5.100 | 0.230 |  |  |
| 3 |  | 0.900 |  |  |
| 4 |  | 0.053 |  |  |
| 4 |  | 0.003 |  |  |
| 5 |  | 1.200 |  |  |
| 6 |  | 0.177 |  |  |
| 7 |  | 0.600 |  |  |
| 8 | 3.900 |  | 0.253 |  |
| 8 |  |  | 0.600 |  |
| 9 | 5.400 |  | 0.600 |  |
| 9 |  |  | 0.300 |  |
| 10 | 3.900 |  | 0.600 |  |
| 10 |  |  | 0.297 |  |
| 11 | 4.200 |  | 0.300 |  |
| 11 |  |  | 0.193 |  |
| 11 |  |  | 0.900 |  |
| 12 | 6.000 |  |  | 2.100 |
| 12 |  |  |  | 5.400 |
| 12 |  |  |  | 2.400 |
| 13 | 3.600 |  |  | 2.400 |
| 13 |  |  |  | 3.600 |
| 13 |  |  |  | 1.500 |
| 14 | 3.600 |  |  | 3.900 |
| 14 |  |  |  | 1.200 |

**Figure 3q source data**

|  | % NOS area/CM area | | | |
| --- | --- | --- | --- | --- |
| litter ID | control | EdnrbKO | cKO(Pax2Cre) | cKO(Wnt1Cre) |
| 1 | 18.113 | 0.000 |  |  |
| 1 |  | 0.046 |  |  |
| 2 | 8.642 | 0.000 |  |  |
| 3 |  | 0.000 |  |  |
| 4 |  | 0.013 |  |  |
| 5 | 8.743 |  | 4.891 |  |
| 5 |  |  | 5.419 |  |
| 6 | 7.2148 |  | 8.562 |  |
| 6 |  |  | 10.490 |  |
| 7 | 11.203 |  | 9.091 |  |
| 7 |  |  | 4.364 |  |
| 8 |  |  | 4.688 |  |
| 8 |  |  | 10.930 |  |
| 9 | 13.529 |  |  | 0.040 |
| 9 |  |  |  | 0.007 |
| 10 |  |  |  | 0.000 |
| 11 | 8.815 |  |  | 0.030 |
| 11 |  |  |  | 0.000 |
| 12 |  |  |  | 0.104 |
| 13 | 9.657 |  |  | 0.000 |
| 13 |  |  |  | 0.000 |
| 14 |  |  |  | 0.000 |
