# Supplementary material for: Lineage-specific intersection of endothelin and GDNF signaling in enteric nervous system development
Source: eLife. 2024 Dec 6;13:RP96424. doi: 10.7554/eLife.96424 (PMC11623925; doi:10.7554/eLife.96424)
Supplement: Figure 3—figure supplement 1—source data 1. [file elife-96424-fig3-figsupp1-data1.docx]

**Figure 3- Figure supplement 1 source data**

|  | Pax2Cre/Rosa26 | | | | | |
| --- | --- | --- | --- | --- | --- | --- |
| colon ID | # neuron | # Pax2Cre (%) | # CGRP (%) | # NOS (%) | # Pax2Cre; CGRP (%) | # Pax2Cre; NOS (%) |
| 1 | 374 | 165 (44.12) | 48 (12.83) |  | 39 (10.43) |  |
| 2 | 361 | 209 (57.89) | 31 (8.59) |  | 27 (7.48) |  |
| 3 | 371 | 169 (45.55) | 27 (7.28) |  | 24 (6.47) |  |
| 4 | 159 | 91 (57.23) | 25 (15.72) |  | 23 (14.47) |  |
| 5 | 178 | 96 (53.93) |  | 49 (27.53) |  | 12 (6.74) |
| 6 | 857 | 439 (51.23) |  | 219 (25.55) |  | 78 (9.10) |
| 7 | 96 | 39 (40.63) |  | 27 (28.13) |  | 10 (10.42) |
| 8 | 498 | 180 (36.14) |  | 142 (28.51) |  | 21 (4.22) |
| 9 | 448 | 176 (39.29) |  | 81 (18.08) |  | 19 (4.24) |
|  |  |  |  |  |  |  |
|  | Wnt1Cre/Rosa26 | | | | | |
| colon ID | # neuron | # Wnt1Cre (%) | # CGRP (%) | # NOS (%) | # Wnt1Cre; CGRP (%) | # Wnt1Cre; NOS (%) |
| 1 | 185 | 70 (37.84) | 17 (9.19) |  | 1 (0.54) |  |
| 2 | 184 | 77 (41.85) | 16 (8.70) |  | 1 (0.54) |  |
| 3 | 198 | 77 (38.89) | 5 (2.53) |  | 0 (0) |  |
| 4 | 173 | 71 (41.04) | 22 (12.72) |  | 1 (0.58) |  |
| 5 | 479 | 176 (36.74) | 35 (7.31) |  | 0 (0) |  |
| 6 | 480 | 175 (36.46) |  | 80 (16.70) |  | 67 (13.99) |
| 7 | 87 | 59 (67.82) |  | 28 (32.18) |  | 23 (26.44) |
| 8 | 458 | 248 (54.15) |  | 122 (26.64) |  | 108 (23.58) |
