# Supplementary material for: Lineage-specific intersection of endothelin and GDNF signaling in enteric nervous system development
Source: eLife. 2024 Dec 6;13:RP96424. doi: 10.7554/eLife.96424 (PMC11623925; doi:10.7554/eLife.96424)
Supplement: Figure 7—source data 1. [file elife-96424-fig7-data1.docx]

**Figure 7i-j source data**

|  | distal midgut | | | | | proximal midgut - caudal half | | | | proximal midgut - proximal half | | | |
| --- | --- | --- | --- | --- | --- | --- | --- | --- | --- | --- | --- | --- | --- |
| embryo | | # GFP^+^  (%) | # Ret^+^  (%) | # GFP^+^/Ret^+^  (%) | # GFP^-^/Ret^+^  (%) | # GFP^+^  (%) | # Ret^+^  (%) | # GFP^+^/Ret^+^  (%) | # GFP^-^/Ret^+^  (%) | # GFP^+^  (%) | # Ret^+^  (%) | # GFP^+^/Ret^+^  (%) | # GFP^-^/Ret^+^  (%) |
| Pax2Cre | 1 | 50  (45.05) | 100  (90.09) | 39  (35.14) | 61  (54.95) | 138  (32.02) | 368  (85.38) | 75  (17.40) | 293  (67.98) | 79  (28.01) | 219  (77.66) | 16  (5.67) | 203  (71.99) |
|  | 2 | 22  (40.74) | 52  (96.30) | 20  (37.04) | 32  (59.26) | 75  (26.60) | 231  (81.91) | 24  (8.51) | 207  (73.40) | 75  (26.60) | 231  (81.91) | 24  (8.51) | 207  (73.40) |
|  | 3 | 28  (35.90) | 77  (98.72) | 27  (34.62) | 50  (64.10) | 59  (27.96) | 183  (86.73) | 31  (14.69) | 152  (72.04) | 27  (21.43) | 107  (84.92) | 8  (6.35) | 99  (78.57) |
|  | 4 | 97  (44.70) | 200  (92.17) | 80  (36.87) | 120  (55.30) | 137  (51.70) | 237  (89.43) | 109  (41.13) | 128  (48.30) | 142  (48.80) | 255  (87.63) | 106  (36.43) | 149  (51.20) |
| Pax2Cre/Ednrb | 1 | 5  (71.43) | 2  (28.57) | 0  (0) | 2  (28.57) | 29  (31.52) | 66  (71.74) | 3  (3.26) | 63  (68.48) | 75  (24.19) | 252  (81.29) | 17  (5.48) | 235  (75.81) |
|  | 2 | 3  (42.86) | 4  (57.14) | 0  (0) | 4  (57.14) | 24  (20.51) | 97  (82.91) | 4  (3.42) | 93  (79.49) | 75  (21.25) | 285  (80.74) | 7  (1.98) | 278  (78.75) |
|  | 3 | 0  (0) | 0  (0) | 0  (0) | 0  (0) | 17  (21.25) | 64  (80.00) | 1  (1.25) | 63  (78.75) | 42  (21.76) | 153  (79.27) | 2  (1.04) | 151  (78.24) |
|  | 4 | 6  (20.69) | 25  (86.21) | 2  (6.9) | 23  (79.31) | 48  (22.02) | 176  (80.73) | 6  (2.75) | 170  (77.98) | 103  (24.41) | 345  (81.75) | 26  (6.16) | 319  (75.59) |
|  | 5 | 5  (29.41) | 12  (70.59) | 0  (0) | 12  (70.59) | 242  (23.73) | 144  (81.36) | 9  (5.08) | 135  (76.27) | 84  (24.28) | 284  (82.08) | 22  (6.36) | 262  (75.72) |
|  | 6 | 0  (0) | 1  (100.00) | 0  (0) | 1  (100.00) | 34  (25.95) | 105  (80.15) | 8  (6.11) | 97  (74.05) | 43  (26.54) | 128  (79.01) | 9  (5.56) | 119  (73.46) |
|  | 7 | 6  (26.09) | 18  (78.26) | 1  (4.35) | 17  (73.91) | 159  (31.93) | 378  (75.90) | 39  (7.83) | 339  (68.07) | * | | | |
|  | 8 | 9  (33.33) | 19  (70.37) | 1  (3.70) | 18  (66.67) | 141  (28.54) | 400  (80.97) | 47  (9.51) | 353  (71.46) | 29  (25.00) | 39  (80.17) | 6  (5.17) | 87  (75.00) |
|  | 9 | 7  (14.89) | 43  (91.49) | 3  (6.38) | 40  (85.11) | 196  (32.94) | 446  (74.96) | 47  (7.90) | 399  (67.06) | * | | | |
| Wnt1Cre | 1 | 118  (64.48) | 141  (77.05) | 76  (41.53) | 65  (35.52) | 125  (54.35) | 197  (85.65) | 92  (40.00) | 10.5  (45.65) | 349  (58.95) | 497  (83.95) | 254  (42.91) | 243  (41.05) |
|  | 2 | 40  (57.97) | 57  (82.61) | 28  (40.58) | 29  (42.03) | 82  (47.40) | 137  (79.19) | 46  (26.59) | 91  (52.60) | 282  (57.43) | 386  (78.62) | 177  (36.05) | 209  (42.57) |
|  | 3 | 197  (62.34) | 230  (72.78) | 111  (35.13) | 119  (37.66) | 221  (61.90) | 250  (70.03) | 114  (31.93) | 136  (38.10) | * | | | |
|  | 4 | * | | | | * | | | | 406  (65.91) | 461  (74.84) | 251  (40.75) | 210  (34.09) |
| Wnt1Cre/Ednrb | 1 | 7  (63.64) | 8  (72.73) | 4  (36.36) | 4  (36.36) | 141  (51.46) | 230  (80.70) | 93  (32.63) | 137  (48.07) | 109  (58.89) | 136  (74.73) | 63  (34.62) | 73  (40.11) |
|  | 2 | 0  (0) | 11  (100.00) | 0  (0) | 11  (100.00) | 100  (13.29) | 205  (88.74) | 74  (32.03) | 131  (56.71) | 81  (52.26) | 130  (83.87) | 56  (36.13) | 74  (47.74) |
|  | 3 | 11  (33.33) | 28  (84.85) | 6  (18.18) | 22  (66.67) | 99  (45.21) | 181  (82.65) | 61  (27.85) | 120  (54.79) | 44  (57.89) | 49  (64.47) | 32  (42.11) | 32  (42.11) |
|  | 4 | 3  (42.86) | 4  (57.14) | 0  (0) | 4  (57.14) | 75  (47.17) | 132  (83.02) | 48  (30.19) | 84  (52.83) | 36  (45.00) | 69  (86.25) | 25  (31.25) | 44  (55.00) |
|  | 5 | * | | | | * | | | | 36  (52.17) | 50  (72.46) | 17  (24.64) | 33  (47.83) |
|  | 6 | 2  (40.00) | 4  (80.00) | 1  (20.00) | 3  (60.00) | 67  (51.94) | 105  (81.40) | 43  (33.33) | 62  (48.06) | * | | | |

*: cell count not included due to partial loss of histological sections or uneven immunohistochemical signals.

**Figure 7q-r source data**

| Wnt1Cre/control | | | | Wnt1Cre/Ednrb | | | |
| --- | --- | --- | --- | --- | --- | --- | --- |
| embryo | # Cre | # pTyr1015 (%) | # pTyr1096 (%) | embryo | # Cre | # pTyr1015 (%) | # pTyr1096 (%) |
| 1 | 13 | 13 (100) |  | 1 | 14 | 9 (64.3) |  |
| 2 | 3 | 2 (66.7) |  | 2 | 3 | 0 (0) |  |
| 3 | 14 | 11 (78.6) |  | 3 | 19 | 9 (47.37) |  |
| 4 | 13 | 12 (92.3) |  | 4 | 5 | 0 (0) |  |
| 5 | 11 | 11 (100) |  | 5 | 16 | 4 (25.0) |  |
| 6 | 23 | 19 (82.6) |  | 6 | 19 | 9 (47.4) |  |
| 7 | 20 | 18 (90.0) |  | 7 | 19 | 9 (47.4) |  |
| 8 | 17 | 15 (88.2) |  | 8 | 1 | 0 (0) |  |
| 9 | 9 | 9 (100) |  | 9 | 8 | 5 (62.5) |  |
| 10 | 3 | 3 (100) |  | 10 | 14 |  | 14 (100) |
| 11 | 24 | 21 (87.5) |  | 11 | 1 |  | 0 (0) |
| 12 | 21 | 20 (95.2) |  | 12 | 0 |  | n/a |
| 13 | 23 | 22 (95.7) |  | 13 | 11 |  | 6 (54.5) |
| 14 | 26 | 23 (88.5) |  | 14 | 2 |  | 2 (100) |
| 15 | 16 |  | 16 (100) | 15 | 5 |  | 5 (100) |
| 16 | 12 |  | 10 (83.3) | 16 | 6 |  | 4 (66.7) |
| 17 | 20 |  | 14 (70.0) | 17 | 4 |  | 4 (100) |
| 18 | 25 |  | 25 (100) | 18 | 2 |  | 2 (100) |
| 19 | 9 |  | 8 (88.9) |  |  |  |  |
| 20 | 8 |  | 7 (87.5) |  |  |  |  |
| 21 | 13 |  | 10 (76.9) |  |  |  |  |
| 22 | 1 |  | 1 (100) |  |  |  |  |
| 23 | 11 |  | 11 (100) |  |  |  |  |
| 24 | 8 |  | 7 (87.5) |  |  |  |  |
